# Health, Wellbeing and Empowerment E-workshops for Mothers of Children with Disabilities: A Non-randomised Comparison Study

**DOI:** 10.1007/s10803-024-06287-5

**Published:** 2024-03-23

**Authors:** Helen M. Bourke-Taylor, Monica Leo, Loredana Tirlea

**Affiliations:** 1 Occupational Therapy Department, School of Primary and Allied Health Care, Faculty of Medicine Nursing and Health Sciences, Monash University, Peninsula Campus, Building G, Level 4, McMahons Road, Frankston, VIC 3199 Australia; 2 Faculty of Health, Arts, and Design, Swinburne University of Technology, PO Box 218, Hawthorn, 3122 Australia

**Keywords:** Mothers, Childhood disability, Mental health, e-Health, Health behaviours

## Abstract

Mothers of children with disabilities can experience compromised health. Targeted interventions require investigation to determine effectiveness. Healthy Mothers Healthy Families (HMHF) is a health, wellbeing and empowerment program that addresses mothers need to protect, and or, recover their own health due to caregiving impacts. This study compared the effectiveness of HMHF e-workshops online compared to no intervention. The HMHF e-workshops were delivered to 290 mothers across the 2020–2022 Covid-19 pandemic and 172 participated in research. The HMHF e-workshops included 3 online 2- hour workshops facilitated by credentialled peer-facilitators, closed online group chat, e-workbook and online learning package. Participants in both groups completed surveys pre and post the workshops (or control) over 8–10 weeks. Mothers who participated in HMHF significantly increased health help seeking behaviours (*p* < .001), and improved mental health and health behaviors over time: health behavior (*p* < .001), positive wellbeing (*p* < .004) and depression (*p* < .001) and stress symptoms (*p* = .005). Compared to controls, HMHF e-workshop participants significantly improved health behaviours (*p* < .001) and self-reported symptoms of depression (*p* = .002) and stress (*p* = .005) over 8–10 weeks. E-workshops were accessible and effective for mothers of children with high care needs and family responsibilities across the COVID-19 pandemic. Compared to no intervention, the HMHF intervention was more effective for improving healthy behaviours and mental health.

## Introduction

There is well established research evidence that e-health, group interventions, led by health professionals and delivered by videoconference, teleconference, or webchat, are effective at improving symptoms of anxiety and depression, emotional distress and positive coping (Currie et al., 2022). Research has not targeted the mental and health concerns of adults with family caring responsibilities, illuminating the need for the development of evidence-based solutions for family carers. Support and effective interventions for one large group of family carers, parents of children with disabilities, are keenly sought, because this cohort are known to experience much higher rates of mental and physical health disparity (Catalano et al., 2018). Disproportionately, mothers both shoulder caring responsibilities and experience greater health impacts within families (Catalano et al., 2018; Masefield et al., 2020).

Healthy Mothers Healthy Families© is a long standing, well-established, evidence-based program with ongoing local availability in areas of Australia, since 2012. Grounded in exploratory research (Bourke-Taylor & Jane, 2018; Bourke-Taylor et al., 2010), the program was developed by an occupational therapist (first author) and numerous collaborators including a women’s health general practitioner and mothers with lived experience. Programs include a self-directed, freely available website, workshops with workbook delivered by health practitioners or mothers, and health coaching delivered by an occupational therapist providing therapy for the child with disability. Health coaching involved specific and tailored, collaboration between therapist and client (mother) to support healthy behaviour change within the realities of complex daily routines and little time secondary to child rearing and caring responsibilities. Substantial evidence now underpins the program for face to face workshop delivery (Bourke-Taylor et al., 2019; Bourke-Taylor et al., 2022) and face to face or telehealth coaching (Bourke-Taylor et al., 2023; Harris et al., 2022). Entirely online HMHF workshops have not previously been delivered or evaluated.

Collectively, the aforementioned evidence for the HMHF programs delivered by health professionals demonstrate statistically significant improvements in physical activity, healthy lifestyle changes (such as healthy eating, stress management, increased time in leisure, health help seeking), improved wellbeing and mental health and improved quality of life for the child and family. Face to face workshops with credentialed facilitators who were mothers of children with disabilities with and without health professional qualifications were also effective (Bourke-Taylor et al., 2021a). In order to manage upscale in Australia, the face to face workshop was converted to an online delivery package early in 2020. This change in program delivery coincided with the start of the COVID-19 pandemic.

Prior to the COVID-19 pandemic, programs that supported the health and wellbeing for mothers of children with disabilities were delivered through a range of mostly face to face delivery methods (Bourke-Taylor et al., 2019, 2022). The home-based impact of containment related to the pandemic and home schooling are known to have been stressful for mothers specifically (Polizzi et al., 2022), and families raising children with disabilities (Baten et al., 2023). Therapy services for children with disabilities transitioned swiftly to online delivery and the role of parents as therapist, teacher and collaborator increased exponentially (Fortin-Bédard et al., 2023). The need for mothers to access their own support for health and wellbeing also increased across this period of time (Babore et al., 2023; Colizzi et al., 2020).

Fully online HMHF workshops delivered solely by trained peer mentors as facilitators (mothers) have not previously been delivered or evaluated. The significance of evaluating fully online delivery is underpinned by improved cost effectiveness of online delivery, improved accessibility for mothers without care replacement or low financial/material resources. The aim of this study was to evaluate the effectiveness of group based HMHF workshops adapted for online only delivery and led by mothers of children with disabilities who were specially credentialled for this project. The research questions were:

1. What is the effectiveness of the HMHF e-workshops, on mothers’ health behaviours, mental health, wellbeing and health-help seeking, when delivered by credentialled mothers, for mothers of children with disabilities, over time?
2. How effective is the HMHF e-workshop package for mothers’ health behaviours, mental health and wellbeing, compared to no intervention (control)?

## Methods

### Design

Ethics approval was received from Monash University Human Ethics Research Committee. (Approval number 10960). The design of this study was a two group (intervention and control) non-randomized control trial with baseline (T1) and post intervention of the online HMHF e-workshop package (T2) measurements being used to assess the effectiveness of the intervention. The control group participants self-allocated to participate in the control group or were allocated if they registered for the HMHF e-workshop package but were unable to attend or intended to attend at a later time. Some control group participants were recruited directly through social media posts or newsletters from the advocacy childhood disability organisation.

### Participants

Recruitment occurred though Kindred, an independent, not-for-profit online peer support organisation for families of children with disability, developmental delays and medical needs.  Kindred had national online reach for mothers meeting the inclusion criteria. Participants were recruited simultaneously for both arms of the study. were eligible for the intervention and the research study if they were mothers or primary caregivers of a child with disabilities and voluntarily registered to attend a HMHF e-workshop. Once mothers registered for a workshop, they received three emails inviting them to participate in the research. Mothers could attend the workshops with no obligation to participate in research. There was no cost for the workshops or payment for participating to mothers. Only mothers who voluntarily provided informed consent were included. Participants were provided with a comprehensive e-workbook for the workshops and access to the Healthy Mothers Healthy Families website. After the three parts of the e-workshop were finished, participants who completed at least one of the workshops, were emailed an invite to complete the post-survey. The control group were emailed an invitation to complete the post survey 8 to 10 weeks after they had completed the pre-survey. Questions about the mother’s health status or health help seeking were not included in the control group survey. Retention of mothers in either arm of the study, who provided informed consent and completed at least one survey, was encouraged through automated emails from the advocacy childhood disability organization and Monash University researchers. Monetary incentives to participate in the study were not offered, although participants with nearly complete data sets were offered gift cards (for food and household items) to complete their participation for the final survey.

**Fig. 1:**
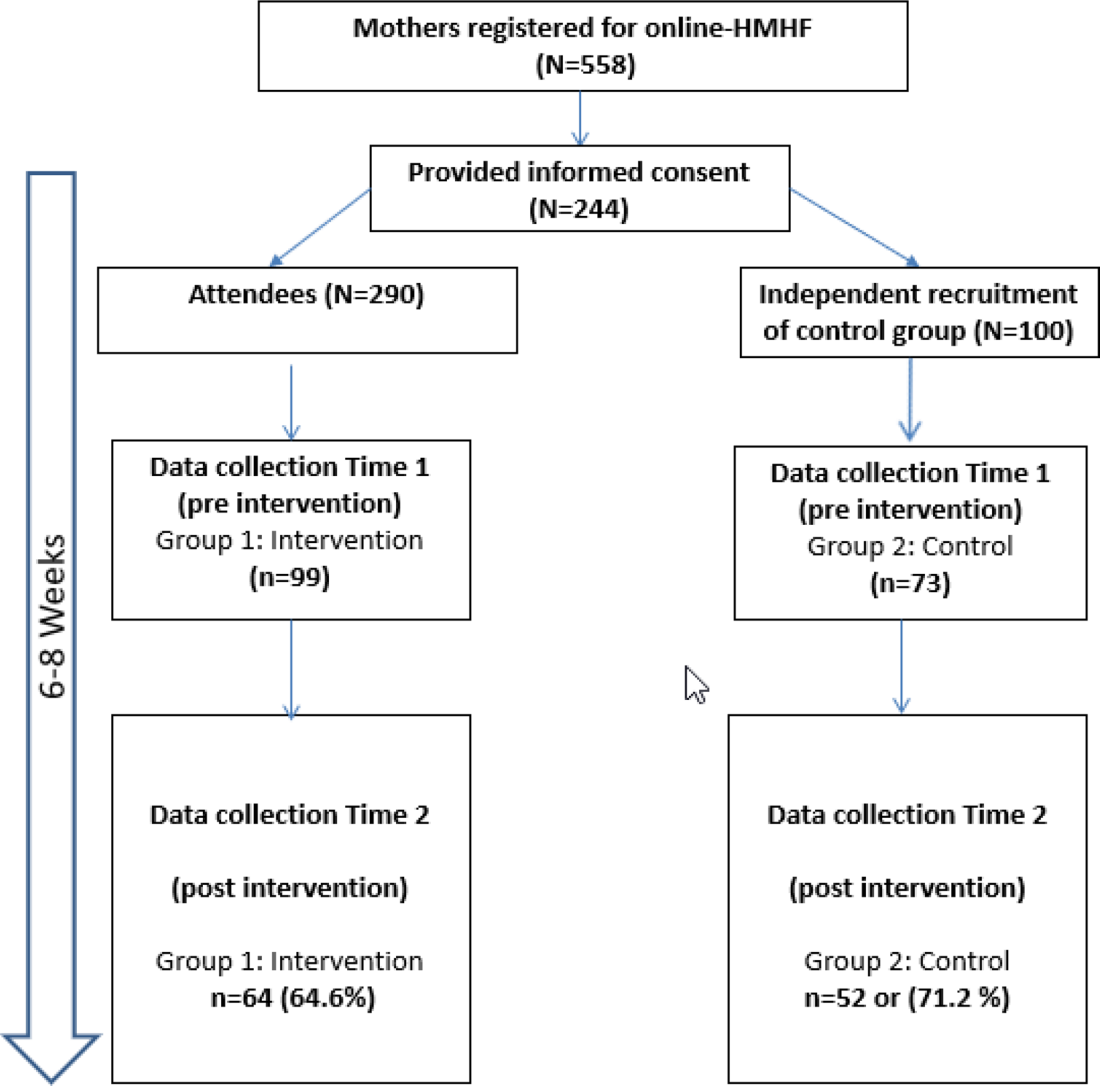
Flow chart indicating the number of potentially eligible participants, those allocated to online intervention and control group, and data collection at T1 and T2 (with attrition percentage)

In total, 559 mothers, registered for the HMHF e-workshops. One hundred and seventy-two mothers provided informed consent to participate: 99 participated in both pre and post surveys and 73 mothers were assigned to the control group (See Fig. 1 for the participants flow in the study). At time 2 attrition rate was 64.6% in the intervention group and 71.2% in the control group (see Fig. 1), thus the attrition rate was slightly higher in the control group.

### The Intervention

The content of the HMHF e-workshop package was based on the already established HMHF intervention described elsewhere (Bourke-Taylor et al., 2022). Collaboration with mothers and the organisation involved in delivery of the e-workshop package contributed to decisions, modifications made to the structure and mode of delivery to make it more accessible. The Template for Intervention Description and Replication (TiDIER) checklist (Hoffmann et al., 2014) was employed to enable a structured way of delivering the content and maintain high fidelity of the core elements of the program across multiple HMHF e-workshops with different facilitators (see Appendix 1). Across eighteen months, 20 cycles of the 3-part e-workshops were delivered by 7 facilitators in pairs.

Healthy Mothers Healthy Families e-workshop intervention consisted of four parts: 6 h (3, 2 h ZOOM workshops delivered over 6 weeks) of facilitated group workshops (between 5 and 20 mothers in each group) that included a 220 slide set with schedule and delivery guide for facilitators; a closed social media group for 8 weeks that included 20 messages aligned with the workshop content and website; online access to a self-paced, 10 module website with embedded video, worksheets, fact sheets and evidence based content; and an e-workbook that hyperlinked to the associated online modules. The workshops included content that has previously been described including the Journey of mothers, health and research findings, stress management approaches, health education, goal setting around health promoting behaviours, support seeking and healthy lifestyle changes or self-defined life goals. Goal setting within the workshops directs women to explore and follow-up with local medical/psychological support if this is required.

The e-workshops were led by trained, credentialed facilitators, requiring about 50 hours of training using the previously tested training package (Bourke-Taylor et al., 2021a). As the workshops progressed, COVID-19 resources and different supports mothers could access were also added to the workshops across the pandemic. Similarly, a 30 min open informal chat session was introduced at the start of every e-workshop to support mothers 3 months into the pandemic. Following the global situation at the time, mothers were dealing with school closures, home schooling, working from home and issues surrounding caring for more vulnerable children. Hence, the timing of many workshops was changed to 8-10 pm at night when children were in bed.

### Outcome Measures

Three outcome measures were used in the study. All outcome measures have been shown to adequately detect change over time in HMHF studies. Internal consistency was excellent for this study (see Table 1).

**Table 1:** Description of instruments and reliability of the measures using Cronbach’s alpha at time 1 and time 2

Construct measured by instrument	Primary outcome measures	Scale description	Time 1 Cronbach alpha	Time 2 Cronbach alpha
Mothers self-reported frequency participating in health promoting activities	Health promoting activities scale (HPAS) Bourke-Taylor et al., (2012)	8-item scale designed to measure the frequency with which a person participates in activities that promote health and wellbeing Respondents estimate the frequency of their participation along a seven-point scale where 1 = never and 7 = once or more every day; higher total HPAS scores reflect greater frequency of healthy behaviours. Higher scores = more frequent participation in health promoting activities	0.79	0.87
Mothers self-reported symptoms of depression, anxiety and stress	Depression Anxiety Stress Scales (DASS 21) Lovibond et al., (1995)	21-item tool used to evaluate mental health symptomology across three subscales: DASS Depression, DASS Anxiety and DASS Stress. Respondents self-rate their symptoms along a 4-point Likert scale (0 = did not apply; 1 = applied to me to some degree or some of the time; 2 = applied to me to a considerable degree or a good part of the time; and 3 = applies very much or most of the time). Higher scores indicate more symptoms of depression, anxiety, or stress	DASS Depression 0.90 DASS Anxiety 0.72 DASS Stress 0.87	DASS Depression 0.91 DASS Anxiety 0.84 DASS Stress 0.89
Mothers self-reported general positive wellbeing	Psychological General Wellbeing Index (positive wellbeing subscale) (PGWBI-PWS) Grossi et al., (2006)	4-item personal wellbeing subscale of the PGWBI is a self-report measure that assesses the wellbeing of adults. Respondents are asked to rate their experiences over the previous month. Four items were included in this study (items 1, 9, 15, 20), including “How have you been feeling in general during the past month?”, How happy, satisfied, or pleased have you been with your personal life during the past month?”, “My daily life was full of things that were interesting to me during the past month”, and “I felt cheerful, light hearted during the past month”. Higher scores = higher personal wellbeing	0.86	0.88

### Health Promoting Activities Scale (HPAS) (Bourke-Taylor et al., 2012)

The HPAS measures the person’s estimation of the frequency with which they participate in health behaviours that promote or maintain health and well-being. The HPAS was developed for mothers of children with disabilities and evaluates the frequency that mothers, with and without children with disabilities, participate in behaviours with known associations with health related quality of life, subjective health, particularly mental health, sleep, obesity and community-based activity. Examples include planning healthy eating and exercise routines, being physically activity alone or with others, taking time out or time socialising with supportive others.

### Depression Anxiety Stress Scale (DASS 21) (Lovibond & Lovibond, 1995)

The DASS includes three self-report scales designed to measure symptoms of depression, anxiety and stress. The Depression scale assesses dysphoria, hopelessness, devaluation of life, self-deprecation, lack of interest/involvement, anhedonia, and inertia. The Anxiety scale assesses autonomic arousal, skeletal muscle effects, situational anxiety, and subjective experience of anxious affect. The Stress scale (items) is sensitive to levels of chronic non-specific arousal. It assesses difficulty relaxing, nervous arousal, and being easily upset/agitated, irritable/over-reactive and impatient. Respondents are asked to use 4-point severity/frequency scales to rate the extent to which they have experienced each state over the past week. The DASS was selected to measure changes to mother’s allocation to groups based on the recommended cut-off scores for conventional severity labels, in the ranges of: normal, mild, moderate, severe or extremely severe.

### Psychological General Wellbeing Index (Positive Wellbeing Subscale) (PGWBI-PWS) (Grossi, et al., 2006)

To measure maternal wellbeing, the Psychological General Wellbeing Index, Positive Wellbeing Subscale (PGWBI-PWS) was extracted and used. The subscale is reliable and valid to use in isolation from the rest of the scale, and added a positive emotional wellbeing aspect to outcomes.

### Descriptive Changes in Help Seeking and Healthy Behaviours

In the pre and post questionnaires, mothers in the intervention group were asked about their frequency of accessing allied health or medical services for themselves (ranging from never = 1 to 2–3 times a month = 4), as well as about intended health behaviour changes prior to the intervention and actual health behaviour changes made following completion of the intervention. Percentage change for these responses was calculated for mothers in the intervention group. The control group post-survey did not contain questions about actual help seeking or changes in health promoting behaviours.

### Data Analysis

Statistical analyses were performed using Excel 2016 and IBM SPSS version 28. Descriptive statistics were computed for all scales and outcome variables of interest. Data measured on continuous scales of measurement were examined for kurtosis and skew to identify potential violations of the assumption of normality of distribution using the Shapiro–Wilk test. The DASS scale and the HPAS scale at time 1 were not normally distributed but, based on previous research, we expected some skewed data particularly for the DASS scale. The mixed designed ANOVA is quite robust and this being the only violation of assumptions and we decided to proceed with parametric analyses.

We conducted preliminary analyses including a Little’s MCAR test to establish if data was missing at complete random or missing systematically (non-random). This indicated that data was missing at complete random. As a result, all data was treated as missing at complete random and listwise deletion was applied to conduct the main analyses. At time 2 loss of data was substantial for all main outcomes (up to 35% missing). To maintain power, we imputed data using the expectation maximization technique (EMT) in SPSS. We conducted all the analyses with the original data and with the complete data set. There was no difference in the main outcome measure overall results between the original data and the imputed data thus for transparency reasons we decided to report the original data results here.

The effect of the online HMHF intervention was evaluated by comparing Groups 1 and 2 across time. Mixed design ANOVA, with time (T1 and T2) as the within-subject variable and group (Groups 1 and 2) as the between- subject variable, were used to detect effects of time, and time × group interactions for each of the outcome measures (DASS, HPAS and Psychological wellbeing). When the interaction was significant, we conducted follow up analyses by splitting the file based on Group to investigate the effects of time for the controls group and the intervention group. Partial eta squared (η^2^) was used to calculate the size of the effect (0.01 = a small effect; 0.06 = medium effect and 0.14 = a large effect. A two tailed alpha significance criterion of 0.05 was used for all the tests.

### Sample Size Calculations

Previous analyses of data from our HMHF coaching pilot non-randomized control trial showed that the effect of the intervention on the participant health promotion activity and behaviour (i.e. Change in HPAS outcome mean score was 5 points and standard deviation was 11). HPAS is the main outcome measures also used in this study. Assuming 80% power, 76 participants per group (N = 152) were required for this study.

## Results

Participants characteristics can be viewed in Table 2. Overall, mothers (N = 172) in the study were, on average, 41 years of age, 56% were married or in a longer-term relationship, 48% had one child, 29% had an undergraduate degree and 33% had a post graduate degree.

**Table 2:** Characteristics of participants at Time 1 (pre intervention) (N = 172)

Characteristics	Participant status, n (%*)	
	Intervention n = 99	Control n = 73
Age (years) Mean (SD) range 24–50 years	40.5 years (5.8)	42.0 (6.8)
Education		
Secondary school	2 (2%)	4 (5%)
Post-secondary education	33 (33%)	27 (37%)
University undergraduate degree	31 (32%)	19 (26%)
University post-graduate degree	33 (33%)	23 (32%)
Relationship status		
Married/living with long term partner	42 (43%)	55 (75%)
Separated/divorced	55 (56%)	13 (18%)
Single	1 (1%)	4 (7%)
No of children in family		
One	61 (63%)	21 (29%)
Two	20 (20%)	34 (46%)
Three	9 (9%)	10 (14%)
Four or more	8 (8%)	8 (11%)
Child disability		
Intellectual disability	5 (5%)	5 (7%)
Autism	54 (55%)	37 (51%)
Downs syndrome	4 (4%)	5 (7%)
ADHD/ADD	23 (23%)	17 (23%)
Anxiety/mental health	15 (15%)	9 (12%)
Cerebral Palsy	5 (5%)	7 (10%)
Other physical	5 (5%)	4(5%)
Developmental or global developmental delay	16 (16%)	11 (15%)
Chronic medical	5 (5%)	0
Genetic disorder	14 (14%)	16 (22%)
Neurological	4 (4%)	9 (12%)
Communication disorder	8 (8%)	1 (1%)
Epilepsy	4 (4%)	5 (7%)
Sensory—hearing or vision	4 (4%)	4 (5%)
Work status (select all that applies)^**^		
Full time	37 (38%)	23 (32%)
Part time	27 (27%)	37 (37%)
Part- or full-time study	38 (38%)	10 (14%)
Voluntary work	8 (8%)	7 (9%)
Unpaid home duties	13 (13%)	19 (26%)
Maternal health		
Reported MH diagnosis	21 (22%)	Not collected
Reported physical health diagnosis	16 (16%)	Not collected
Reported MH and physical health diagnoses	11 (11%)	Not collected
Health-help seeking		
Frequency seeking medical/allied health help		
Never (1)	9 (9%)	Not collected
2–3 times per year (2)	42 (45%)	Not collected
Once per month (3)	20 (21%)	Not collected
2–3 times per month (4)	25 (25%)	Not collected
Mothers indicate ‘yes’ to the type of change they hoped to achieve after the workshop (n = 99)**		
Changes to my mental health and wellbeing	87 (88%)	Not collected
Changes to the way I manage stress in my daily life	62 (63%)	Not collected
Changes to my family’s leisure routine	42 (42%)	Not collected
Changes to the way we manage stress in my family	52 (53%)	Not collected
Changes to the people that I spend time with	21 (21%)	Not collected
Changes to my leisure routine and participation in healthy activity	43 (43%)	Not collected
Changes to my physical activity	48 (49%)	Not collected
Changes to my diet	41 (41%)	Not collected
Changes to how I view myself	49 (64%)	Not collected
Changes to my sleep quality	45 (46%)	Not collected
Other change^*^	15 (22%)	Not collected

Although there were some differences in demographics between the intervention and control groups at baseline there were no significant differences in scores for the outcome measures of interest

^*^Percentages taken to full number, no decimal places and therefore may add up to 101 or 99

^**^Percentages do not add up to 100% as select all that apply

Independent sample t tests were first conducted to assess any differences at baseline between the control and the intervention groups for all outcome variables of interests including: the HPAS; the DASS scale (three subscales- DASS-Stress, DASS-Depression and DASS-Anxiety) and the PGWBI-PWS. At baseline, there were no statistically significant differences between the intervention and the control group on any outcome measures (see Table 3).

**Table 3:** Outcome variables scores at Time 1 (T1) for intervention and control groups

Outcome variable	Control			Intervention			*t, df, p*	95% CI upper, lower
	N	M	SD	N	M	SD		
T1 HPAS	73	23.58	10.18	96	22.33	7.58	*t* (167) = 0.91, *p* = 0.364	1.45, 3.94
T1 DASS Depress	73	6.16	5.39	93	7.37	4.97	*t* (165) = 1.50, *p* = 0.068	0.38, 2.50
T1 DASS Anxiety	73	7.26	6.40	90	6.84	7.01	*t* (161) = 0.39, *p* = 0.696	1.68, 2.51
T1 DASS Stress	72	19.42	10.56	94	21.32	9.17	*t* (164) = 1.24, *p* = 0.217	1.13, 4.93
PGWBI-PWS	73	7.77	3.74	99	7.47	3.23	t (170) = 0.55, p = 0.584	0.76, 1.34

Health Promoting Activity Scale (HPAS), Depression, Anxiety, Stress Scales (DASS) and Psychological General Wellbeing Index – psychological wellbeing subscale (PGWBI-PWS)

We conducted mixed designs ANOVAS to explore changes in scores (pre and post participation in the online HMHF intervention) across the primary outcome variables of interest. We expected that scores would improve on these outcome variables for the participants in the intervention and scores would stay relatively the same for the control group participants.

The scores for the DASS subscales were calculated and cut-off scores derived for the intervention group at before and after the HMHF e-workshops. Changes in the number of participants in the cut of categories based on DASS-depression/anxiety/stress severity can be viewed in Table 4.

**Table 4:** Summary of Intervention group mothers’ mental health status pre (T1) and post (T2) HMHF intervention

Depression anxiety stress scale scores	Participant status, n (%*) T1	Participant status, n (%*) T2
Depression subscale scores mean (SD)	n = 95	n = 61
	7.37 (SD = 4.97)	4.95 (SD = 4.95)
Normal	63 (66.02%)	48 (78.69%)
Mild	19 (20.21%)	8 (13.11%
Moderate	11 (11.70%)	5 (8.20%)
Severe	1 (1%)	0
Extremely severe	1 (1%)	0
Anxiety subscale scores mean (SD)	n = 90	n = 60
	6.84 (SD = 7.01)	5.47 (SD = 6.65)
Normal	57 (63.33%)	44 (73.33)
Mild	11 (12.22%)	3 (5.00%)
Moderate	11 (12.22%)	7 (11.67%)
Severe	3 (3.33%)	3 (5.00%)
Extremely severe	8 (8.89%)	3 (5.00%)
Stress subscale scores (n = 65) mean (SD)	n = 94	n = 62
	21.32 (SD = 9.17)	15.61 (SD = 9.10)
Normal	26 (27.66%)	34 (54.84%)
Mild	16 (17.02%)	9 (14.52%)
Moderate	21 (22.2%)	11 (17.74%)
Severe	19 (15.4%)	4 (6.45%)
Extremely severe	12 (12.77%)	4 (6.45%)

### Results Post-HMHF-Health Promoting Activities Scale

**Fig. 2:**
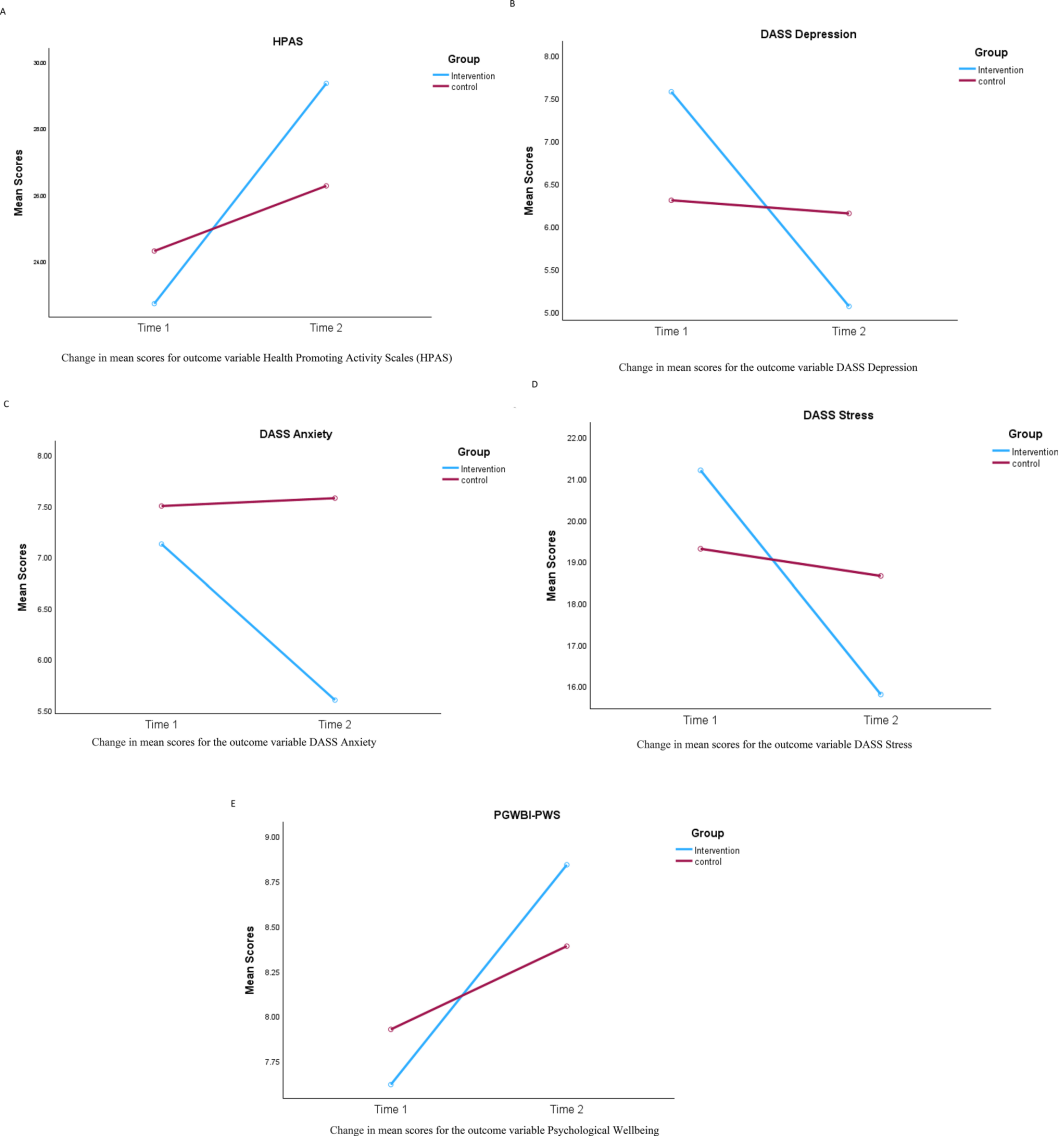
Graphs showing change in mean scores for outcome variables health promoting activity scales (HPAS), depression, anxiety, stress scales (DASS) and personal wellbeing index—psychological wellbeing scale (PWBGI-PWS) for intervention and control groups at time 1 and time. **A** Change in mean scores for outcome variable health promoting activity scales (HPAS). **B** Change in mean scores for the outcome variable DASS depression. **C** Change in mean scores for the outcome variable DASS anxiety. **D** Change in mean scores for the outcome variable DASS stress. **E** Change in mean scores for the outcome variable psychological wellbeing

A mixed design analysis of variance was conducted on the HPAS data. This analysis revealed a significant increase in mean HPAS score across time, *F* (1,109) = 36.13, *p* < 0.001, *partial η*^2^ = 0.250. However, as can be seen in Fig. 2, this increase is more apparent for the intervention group. The interaction between time and group is significant, *F* (1,109) = 10.67, *p* = 0.001, *partial η*^2^ = 0.089. Follow up analyses suggested that for the control group, there is no significant main effect for time, *F*(1,51) = 3.63, *p* = 0.063 whilst for the intervention group there is a significant increase for the health promoting activity from baseline to 6 weeks, *F*(1,59) = 45.68, *p* < 0.001 *partial η*^2^ = 0.432. Furthermore, the intervention group mean reported a 6.62-point change, which is greater than the minimum detectable change (MDC = 5) for this tool (Muskett et al., 2017).

### Results Post-HMHF-DASS Depression

A mixed design analysis of variance was conducted on the DASS Depression data. This analysis revealed a significant decrease in mean depression score (DASS-depression) across time, *F* (1,109) = 12.24, *p* < 0.001, *partial η*^2^ = 0.101. However, as can be seen in Fig. 2, this decrease is more apparent for the intervention group. The interaction between time and group is significant, *F* (1,109) = 9.58, *p* = 0.002, *partial η*^2^ = 0.081. Follow up analyses suggested that for the control group, there is no significant main effect for time, *F*(1,51) = 08, *p* = 0.784 whilst for the intervention group there is a significant decrease for the depression score from baseline to 8–10 weeks, *F*(1,58) = 23.40, *p* < 0.001 *partial η*^2^ = 0.287.

### Results Post-HMHF-DASS Anxiety

A mixed design analysis of variance was conducted on the DASS Anxiety data. This analysis revealed although there was a decrease in mean anxiety score (DASS-anxiety) across time (see Fig. 2), this was not significant *F*(1,105) = 1.63, *p* = 0.204. Furthermore, there was no significant interaction between time and group *F*(1,105) = 1.99, *p* = 0.204.

### Results Post-HMHF-DASS Stress

A mixed design analysis of variance was conducted on the DASS Stress data. This analysis revealed a significant decrease in mean stress score (DASS-Stress) across time, *F* (1,110) = 13.63, *p* < 0.001, *partial η*^2^ = 0.110. However, as can be seen in Fig. 2, this decrease is more apparent for the intervention group. The interaction between time and group is significant, *F* (1,110) = 8.38, *p* = 0.005, *partial η*^2^ = 0.071. Follow up analyses suggested that for the control group, there is no significant main effect for time, *F*(1,51) = 0.36, *p* = 0.552 whilst for the intervention group there is a significant decrease for the stress score from baseline to 8–10 weeks, *F*(1,59) = 20.31, *p* < 0.001 *partial η*^2^ = 0.256.

### Results Post-HMHF-PGWBI-PWS

We conducted a mixed designed ANOVA on the PGWBI-PWS data. This revealed a significant increase in mean PWB scores across time F (1,115) = 8.54, *p* = 0.004, partial *η*^2^ = 0.069. For the intervention group there is a significant increase for psychological wellbeing from baseline to 6 weeks, F(1,62) = 10.83, *p* = 0.002 partial *η*^2^ = 0.149 (See Fig. 2). The interaction between time and group was not significant, F (1,115) = 1.73, *p* = 0.191).

### Changes in Frequency of Seeking Health Services and Healthy Behaviour Changes

A paired sample *t*-test revealed there was a significant change (*t*(113) = 5.65, *p* < 0.001) on average in the frequency of service access and help seeking following participation in the HMHF program (before program, *M* = 1.89, *SD* = 1.60) and (after the program, *M* = 2.83, *SD* = 1.05). This change represented a shift in health help-seeking from between never and 2–3 times per month to 2–3 times per month and once a month.

Actual health behaviour changes 8–10 weeks after attendance at the workshop were calculated as percentage agreement with items for the intervention participants (*N* = 99). From highest to lowest percentage change (n = 99), results were: “Changes to my mental health and wellbeing” (*n* = 31, 31.3%);“Changes to how I view myself” (*n* = 31, 32%); “Changes to my physical activity” (*n* = 30, 30.3%); “Changes to my diet” (*n* = 23, 23.2%); “Changes to the way we manage stress in my family” (*n* = 21, 21.2%).“Changes to my leisure routine and participation in healthy activity” (*n* = 21, 21.2%); “Changes to the people that I spend time with” (*n* = 19, 19.2%); “Changes to the way I manage stress in my daily life” (*n* = 13, 13.1%); and “Changes to my family’s leisure routine” (*n* = 12, 12.1%).

## Discussion

Mothers significantly improved their health behaviours, mental health and wellbeing, and use of health services following participation in the HMHF e-workshop package. Aligned with the in-person workshops, the online workshops effectively reduced mothers’ depressive and stress symptoms. Furthermore, mothers also increased their participation in health promoting activities, demonstrating a more significant increase in the frequency of participation in health promoting activity than mothers in the in-person group (Bourke-Taylor et al., 2022). This is congruent with other research that suggests that online formats can be as effective as in-person formats or a combination of both (Lau et al., 2022). Recent research continues to demonstrate the inextricable relationship between maternal participation in health promoting activities and the participation of children with disabilities (Wang et al., 2023).

When participants in the in-person HMHF workshops were compared to mothers in the current study, the largest group were mothers of a child with ASD, which is congruent with the incidence of ASD. Further, compared in in person delivery, the online HMHF workshops attracted much larger percentages of single mothers (57% vs 15%), employed mothers (65% vs 43%) and one child families (71% vs 37%) (Bourke-Taylor et al., 2022). These findings indicate that the e-workshops are an effective delivery method and have the potential to reach a different subsection of mothers who are not partnered, work more and have a single child. It is imperative that health professionals supporting families consider alternative formats and intervention points that cater for the diverse needs of different mothers.

Mothers of children with disabilities have high mental health support needs and face a wide range of barriers to access services for themselves (Bradshaw et al., 2019; Gilson et al., 2021). Programs and interventions targeting the complex needs of mothers must take into consideration flexible service delivery formats to increase accessibility for mothers who find it difficult to seek out these services (Gilson et al., 2017). The effective HMHF e-workshop intervention employed a multi-component, comprehensive approach to delivery of the intervention. Participants not only engaged in the videoconferencing group sessions, but were also provided with a detailed eBook, direction to the HMHF website and Messenger chat posts regularly throughout the 6-weeks of the intervention. Incorporating multiple modalities provided options for mothers to interact with the information in different ways and at times that were convenient for them. Chafouleas et al. (2020) completed interviews with primary caregivers of children with developmental disabilities that reinforced that carers experience differing support needs and a wide range of barriers related to both caregiving, personal factors and variations in their child’s condition and trajectory, and highlighted the requirement for programs to account for this in order to reach more mothers. In the current study, mothers increased their health help seeking and followed up with medical and allied health appointments. These findings suggest that mothers became empowered to navigate barriers to their own service use, thereby further reinforcing the need for similar programs to coach mothers towards accessing services for themselves.

The HMHF e-workshop program also provided direct peer support through the trained peer mentors facilitating the online group sessions. Support from peers was identified as important by parents in a critical interpretative synthesis of interventions targeting mental health of parent carers of children with ASD in reducing isolation, normalisation and validation of the parent carer experience (Catalano et al., 2018). Further, the study recommended direct communication approaches (i.e. same time video as used in the current study) as opposed to indirect communication (Catalano et al., 2018). Other studies including Sartore et al. (2021), found that the effectiveness of peer support for informal interventions is inconclusive, although parents valued these interventions. Further robust research that provides detailed information on credentialing of peer mentors is required to better understand the contribution of this to supporting maternal health and wellbeing.

A recent systematic review of interventions for mental health, wellbeing and stress for mothers of children with disabilities, revealed that numerous studies excluded mothers with known mental health conditions, or mothers who had sought supports for mental health in the previous year (Bourke-Taylor et al., 2021b). Additionally, mothers tended to under-report and were less likely to seek out services for themselves, perceiving their needs to be less important than those of their families (Gilson et al., 2017, 2021). Mothers with mental health conditions were not excluded in the current study and results highlight that these mothers made improvements in their health self-management and mental health. On the DASS Stress subscale, approximately 27% of mothers recorded scores in the severe or extremely severe range pre the intervention, and this had reduced to approximately 12% after. Mothers who scored in the ‘Normal’ ranges on the Depression subscale increased from 66 to 79%, on the anxiety scale scores in the normal range increased from 63 to 73% and on the Stress scale they increased from 28 to 55%. Future research including mothers with known mental health conditions is important in evaluating effectiveness of interventions to understand optimal pathways of providing services that meet their needs, given they are pivotal in ensuring outcomes for their children.

One limitation of this study is the non-random allocation of participants to the intervention or control group for the study. This common issue in the analysis of a non-random allocated trial design is that it fails to control for differences between participants. In the current study for the primary outcome measures, we found no significant differences between the control and the intervention participants at baseline. Hence, random allocation of control and intervention was somewhat mitigated. Regardless, some unmeasured differences between groups may have impacted results. For example, the motivation of women allocating to the intervention arm cannot be measured and therefore cannot be discounted from contributing to outcomes. Similarly, with regard to the control group, the better life situation or access to supports may have implicated this group to believe that they did not need a health and wellbeing intervention, thereby also impacting their own lack of change. Future studies could randomise recruitment points or services in order to achieve a more rigorous study design such as a cross over designs to enable participants in the control group later access to the intervention.

Drop-out rates in the present study were similar to those reported in randomized controlled trials for pediatric chronic conditions longitudinal studies i.e. mean attrition rate was 20% (range 0–54%) for initial follow-up and 32% (range 0–59%) for extended follow-up (Karlson & Rapoff, 2008). Nevertheless, a limitation of the current study was the loss of missing data at follow up. In a future study this should be taken into consideration and best efforts should be made to recruit a larger sample size.” Other limitations include that there was no long-term data collected for the mothers who received the intervention and the study included mothers of children with a wide range of disabilities ad different life situations. In the current study, the HMHF e-workshop intervention improved mothers’ ability to make actual changes to increase their participation in healthy behaviours. Collection of follow-up data would be beneficial in understanding if mothers are able to sustain the uptake of these behaviours which is of particular importance given the enduring nature of their caregiving role.

Whilst there has been some research into parenting interventions for children with ASD, many of these were parent training interventions and the evidence on effectiveness of parent support interventions is inconclusive due to significant heterogeneity in interventions and variables measured (MacKenzie & Eack, 2022). Further research into mothers with children with different disabilities, in different regions and cultures would add to the body of evidence on best practice for supporting this highly vulnerable group.

The current study provides evidence that HMHF e-workshop delivery is as effective as in person delivered workshops, when coupled with the same suite of resources (work books, website etc.). Supporting children with disabilities necessitates active, evidence informed interventions and systems to support the mental and overall health of parents who provide intense day to day care (Miodrag et al., 2015). Parents are likely to provide care over several decades, making maintaining a parent/carers capacity to care a responsibility for the family-focused paediatric allied health or medical practitioner (Chambers & Chambers, 2015). Family health behaviours inside the home and healthy activities in the community outside of the home are greatly impacted by both the health status of parents and the needs of children/young people with disability (Ranger et al., 2021). HMHF programs are an example of one effective option that has been available to Australian mothers in various forms for over a decade. The development and evaluation of programs such as HMHF should continue to be a priority in the delivery of childhood disability services internationally.

## Appendix

See Table 5.

**Table 5:** Template for Intervention Description and Replication, (TIDieR) Criteria Applied to the Healthy Mothers Healthy Families, e-workshops plus social media closed group messages in this research

BRIEF NAME	
Provide the name or a phrase that describes the intervention	Healthy Mothers Healthy Families e-workshops
WHY	
Describe any rationale, theory, or goal of the elements essential to the intervention	HMHF is a program that seeks to empower and resource mothers of children with a disability to optimise their health and healthy behaviors through a friendly relatable website with 18 videos of other mothers and professionals; dissemination of research evidence about the health and situation of mothers including ways in which health behaviors are changeable, health coaching, goal-setting, health education, and psychoeducation The objectives of the HMHF intervention were to: ⋅Provide women with the opportunity to learn about research relating to the impact of caring for children with a disability or severe chronic medical condition ⋅Educate women about healthy lifestyle habits ⋅Contribute to the prevention of women’s health problems that may be related to caring ⋅Assist mothers to identify challenges and strengths within their unique life situation ⋅Assist women to determine desired life goals and master strategies to better health and well being ⋅Provide a range of education opportunities to help improve resilience, communication skills, manage stress and stay strong ⋅Connect mothers with local supports, organisations, recreational opportunities that assist them in their daily responsibilities ⋅Enable skills to set goals around health, well-being and participation in social and community activities and set an action plan ⋅Facilitate development of effective self and family advocacy skills ⋅Facilitate help seeking and use of available formal and informal supports within the health system and the community
WHAT	
Materials: Describe any physical or informational materials used in the intervention, including those provided to participants or used in intervention delivery or in training of intervention providers. Provide information on where the materials can be accessed (e.g. online appendix, URL)	1. Specifically, designed workbook 2. Access to 24 fact sheets; 3. 18 videos; Specialist input from other suitably qualified health professionals: ⋅General practitioner ⋅Occupational therapist ⋅Physiotherapist ⋅Dietitian ⋅Counsellor Specialist input from other mothers with lived experience 4. Comprehensive website to be completed as desired by mothers 5. Online HPAS quiz All information can be accessed via the Healthy Mothers Healthy Families website (https://healthymothers-healthyfamilies.com/)
Procedures: Describe each of the procedures, activities, and/or processes used in the intervention, including any enabling or support activities	
	1.Individualised HMHF-credentialling for new peer mentor facilitators 2. E-workshop and Messenger posts delivered The program was delivered via 3 × e-workshops (over 6 weeks), coupled with 20 Messenger posts following each session with content directly related to topics covered that session as per outline below: Start–Messenger posts (unique to the online workshops.) to mothers about the upcoming workshops Workshop 1—2 h Messenger posts about workshop 1 and the online package Workshop 2—2 h Messenger posts about workshop 2 and the online package Workshop 3—2 h Messenger posts about workshop 3 and the online package–End Posts promoted interaction with the HMHF website and prompts regarding health promoting activities. Content in the Messenger posts covered topics such: ⋅The journey of mothers, linking your health with the journey and sharing the journey with other mothers ⋅Increasing physical activity in family routines ⋅Increasing self-care activities and leisure time in routines ⋅Small changes activity ⋅Celebrating small changes ⋅Ways to manage emotional wellbeing and stress ⋅Identifying supports and bringing them around you ⋅Reflecting on your own health needs ⋅Creating time for yourself 3. Self-managed component Access to a specially designed website with a 10-module online learning package: Module 1: The Journey of Mothers Module 2: Health and Research Findings Module 3: What Mothers Say About Stress Module 4: Healthy Mind, Healthy Mother Module 5: Active Healthy Mother Module 6: Healthy Eating Module 7: Bringing support around you and your family Module 8: Managing healthy home routines and having fun in the community Module 9: Time for me planning Module 10: Managing and staying strong
WHO PROVIDED	
For each category of intervention provider (e.g. psychologist), describe their expertise, background and any specific training given	Seven peer mentor mothers were trained and credentialled as facilitators for the online-HMHF program. Three of these had been previously trained to delivery HMHF f2f program Facilitators were mothers with lived experience of having children with disabilities Peer mentor facilitators completed; ⋅2 × half day educational workshops with first author covering foundation knowledge of unique situation of mothers of children with disability and HMHF program ⋅8-h professional training course included in the HMHF website and a 2-h session with first author to ask any questions related to this ⋅3 × 2.5-h ZOOM training sessions with first author to learn the content and delivery of parts 1, 2 and 3 of the online HMHF workshops ⋅Co-facilitated workshops with a credentialled facilitator ⋅Received feedback from workshop participants and first author
HOW	
Describe the modes of delivery (e.g. face-to-face or by some other mechanism, such as internet or telephone) of the intervention and whether it was provided individually or in a group	E-workshops combined with self-paced online content E-workshops were delivered via ZOOM platform and run at a range of different times of day (daytime and evenings) to accommodate needs of mothers
WHERE	
Describe the type(s) of location(s) where the intervention occurred, including any necessary infrastructure or relevant features	Videoconferencing and web-based Facebook Messenger posts
WHEN and HOW MUCH	
Describe the number of times the intervention was delivered and over what period of time including the number of sessions, their schedule, and their duration, intensity or dose	3 × 2-h online workshops 20 Facebook Messenger posts
TAILORING	
If the intervention was planned to be personalised, titrated or adapted, then describe what, why, when, and how	Intervention is administered similarly for mothers. Flexibility exists in terms of length of time interacting with online modules
MODIFICATIONS	
If the intervention was modified during the course of the study, describe the changes (what, why, when, and how)	Responsive changes were made to include COVID-19 resources and different supports across the pandemic Timing of all workshops was changed to 8-10 pm at night when children were in bed during the pandemic
HOW WELL	
Planned: If intervention adherence or fidelity was assessed, describe how and by whom, and if any strategies were used to maintain or improve fidelity, describe them	Fidelity assured through equivalent training of peer mentor facilitators Fidelity supported by consistent website—no changes to occur across 12-week period First author available during business hours for any questions by peer mentor facilitators
Actual: If intervention adherence/fidelity was assessed, describe the extent to it was delivered as planned	A high level of adherence and fidelity was implemented. All facilitators who delivered the intervention used the tools provided and adhered to a supportive positive interaction allowing mothers to identify their own solutions. The content was guided by the same tools (website, workbook, fact sheets etc.)
